# Cat8 Response to Nutritional Changes and Interaction With Ehrlich Pathway Related Factors

**DOI:** 10.3389/fmicb.2022.898938

**Published:** 2022-06-15

**Authors:** Zhengda Du, Hong Deng, Yanfei Cheng, Zhiguang Zhai, Xuena Guo, Zhaoyue Wang, Xiuping He

**Affiliations:** ^1^CAS Key Laboratory of Microbial Physiological and Metabolic Engineering, State Key Laboratory of Mycology, Institute of Microbiology, Chinese Academy of Sciences, Beijing, China; ^2^College of Life Sciences, University of the Chinese Academy of Sciences, Beijing, China; ^3^Institute of Basic Theory for Chinese Medicine, China Academy of Chinese Medical Sciences, Beijing, China

**Keywords:** Cat8, Snf1, Ehrlich pathway, RNA-seq, ChIP-seq, Aro80, Gat1

## Abstract

Cat8 is an important transcription factor regulating the utilization of non-fermentative carbon sources in *Saccharomyces cerevisiae*. However, our previous studies found that Cat8 may play a critical role in nitrogen metabolism, but the regulatory mechanism has not been elucidated. In this study, the nuclear localization and analysis of regulatory activity showed that the Cat8 function relies on Snf1 kinase. In the fermentation with glucose or glycerol as carbon sources under phenylalanine (Phe) induction, by comparing the changes of cellular gene expression and Cat8 target gene binding profiles after Cat8 overexpression, enhanced transcription was shown among key genes involved in the Ehrlich pathway (e.g., *ARO9*, *ARO10*, and *ADH2*) and its upstream and downstream related factors (e.g., *GAP1*, *AGP1*, *GAT1*, *PDR12*, and *ESPB6*), indicating that Cat8 participated in the regulation of nitrogen metabolism. Moreover, highly active Cat8 interacts with transcriptional activator Aro80 and GATA activator Gat1 coordinately to regulate the transcription of *ARO10*. Altogether, our results showed that Cat8 may act as a global transcription factor in response to nutritional changes, regulating both carbon and nitrogen utilization. This provides a new insight for us to explore the regulation of cell nutrient metabolism networks in yeast.

## Introduction

Cat8, a zinc cluster transcriptional activator, is necessary for regulating gene expression during yeast diauxic shift from fermentative metabolism to respiratory metabolism (Haurie et al., 2001). Cat8 activates many metabolic pathways such as gluconeogenesis, tricarboxylic acid (TCA) cycle, the glyoxylate cycle, β-oxidation of fatty acids, and respiratory chain to enable cells to utilize non-fermentative carbon sources (Hedges et al., 1995; Bonander et al., 2008; Laera et al., 2016). The expression and transcriptional activity of Cat8 are regulated complicatedly: In the presence of glucose, the transcription factor Mig1 (C_2_H_2_ zinc finger protein) becomes dephosphorylated and localizes to the nucleus, repressing the expression of *CAT8*. When glucose is depleted, the Snf1 kinase complex (AMP-activated S/T protein kinase) is activated and phosphorylates Mig1 to remove the repression of Mig1, allowing *CAT8* to initiate transcription [reviewed in Zaman et al. (2008)]. In addition, Cat8 is controlled by the carbon source at a post-transcriptional level, which undergoes multi-step phosphorylation before activating the transcription of target genes (Randez-Gil et al., 1997), among them, the Snf1 kinase complex plays a dominant role in the phosphorylation of Cat8. However, the regulatory mode of Cat8 phosphorylation and related kinases are still unclear (Turcotte et al., 2010). It has been reported that the Cat8 middle homology region (MHR) may harm the transcriptional activation domain (TAD) (Supplementary Figure 1; Delaveau et al., 1994; Schjerling and Holmberg, 1996), and phosphorylation of Cat8 by kinases appears to relieve this negative effect (Charbon et al., 2004). Furthermore, overexpression of Cat8 under repressive conditions (high glucose, 2%) increases the expression of CSRE (carbon source-responsive element)-dependent genes, but the transcriptional activity of Cat8 is still affected by the activity of the carbon source-controlled TAD (Hedges et al., 1995; Rahner et al., 1996, 1999). The post-translational modification of Cat8 regulated by carbon source produces different degrees of activation of its transcriptional activity (Charbon et al., 2004), which may be the reason why yeast can perform refined transcriptional reprogramming in response to different types of carbon sources. Collectively, Cat8 transcription and transcriptional activity co-regulate cellular responses to changes in carbon sources in the environment.

Cat8 is an important transcription factor for the response to the carbon source, some researchers ever predicted Cat8 and Mig1 as key transcriptional regulators controlling the differential expression of the genes affected by aromatic alcohol communication (Wuster and Babu, 2010). Aromatic alcohols 2-phenylethanol (2-PE) and tryptophol are quorum-sensing signaling molecules for fungal filamentous growth (Chen and Fink, 2006), which are synthesized from Phe and tryptophan (Trp) in a three-step reaction of transamination, decarboxylation, and reduction via Ehrlich pathway (Etschmann et al., 2002). Up to now, the regulation mechanism of the Ehrlich pathway is not yet very clear and only a few regulatory factors have been reported. It has been reported that the transcription factors Aro80 and GATA activators (Gln3 and Gat1) are involved in the regulation of the Ehrlich pathway in *Saccharomyces cerevisiae* (MacPherson et al., 2006; Eden et al., 2007; Lee and Hahn, 2013). In our previous study on the regulation of 2-PE synthesis, we found that Cat8 could promote yeast cells to synthesize 2-PE, moreover, Cat8 overexpression strain in Phe medium significantly up-regulated the expression of key enzymes in the Ehrlich pathway (Wang et al., 2017), suggesting that Cat8 is not only significant in carbon metabolism regulation but also may play an essential role in regulating the Ehrlich pathway and nitrogen source metabolism.

For a deeper understanding of Cat8 role in nitrogen metabolism response, we carried out the investigation on the differences between the expression, functional activity, and regulation of Cat8 under two conditions of fermentation and respiration in yeast in response to nitrogen source metabolism. Our results suggest that highly active Cat8 bound to the promoter region and enhanced the transcription of key genes involved in the Ehrlich pathway and its upstream and downstream related genes. Moreover, Cat8 interacts with transcriptional activator Aro80 and GATA activator Gat1 coordinately to regulate the transcription of *ARO10*. Altogether, the data support the view that Cat8 is involved in the regulation of nitrogen source metabolism.

## Materials and Methods

### Media and Yeast Cultivation Conditions

Yeast cells were grown in a standard YPD or SC medium at 30°C. The solid medium contains 20 g/L agar. YPD medium consisted of 10 g/L yeast extract, 20 g/L peptones, and 20 g/L glucose. The defined synthetic complete (SC) medium consisted of 6.7 g/L yeast nitrogen base (YNB), 20 g/L carbon sources (glucose or glycerol), and 20 mg/L uracil (Ura), and 40 mg/L each of Trp, leucine (Leu), and histidine (His). The fermentation medium is obtained by adding 2 g/L Phe to the SC medium containing 20 g/L glucose (Glc) or glycerol (Gly).

### Strain Construction

Yeast strains used in this work are derived from *S. cerevisiae* YS58 and listed in Supplementary Table 1. The *S. cerevisiae* YS58 strain was kindly given by Prof. Teunissen and was derived from diploid yeast YS60 (Teunissen et al., 1993). All DNA fragments integrated into the genome were done by the method of homologous recombination. The 3 × HA tag was connected to the carboxyl terminus of Cat8 by introducing Zeocin resistance to obtain the YS58-HA strain, and the promoter of *CAT8* was replaced with the *TEF2* promoter by introducing G418 resistance to obtain YS58-CAT8-HA strain. Based on YS58-HA and YS58-CAT8-HA strains, YS58-HA Δsnf1 and YS58-CAT8-HA Δsnf1 strains were obtained by introducing hygromycin resistance and deleting *SNF1*. The following strains with gene deletion were obtained using the scarless knockout method created by our laboratory (Zhou et al., 2021), including the strains YS58-Δcat8, YS58-Δaro80, and YS58-Δgln3gat1. *CAT8* self-promoter was further replaced with *TEF2* promoter by the seamless replacement method to obtain YS58-CAT8 Δaro80 and YS58-CAT8 Δgln3gat1 strains. The YCpA-CAT8-GFP plasmid was introduced into YS58-Δcat8 to obtain the CAT8-EGFP strain. CAT8-EGFP Δsnf1 strain was then obtained by using hygromycin to knock out *SNF1* in the CAT8-EGFP strain. Plasmids YEpA-ARO80-Myc, YEpA-GLN3-Myc, and YEpA-GAT1-Myc were introduced into the YS58-CAT8-HA strain, respectively, then YS58-CAT8-HA Aro80-Myc, YS58-CAT8-HA Gln3-Myc, and YS58-CAT8-HA Gat1-Myc strains were obtained.

### Gene Cloning and Plasmids Construction

Plasmids used in this study were listed in Supplementary Table 2. YCpA-CAT8-EGFP backbone was derived from pYC-AGA constructed in our laboratory. The enzyme digestion at the *Bam*HI site linearized pYC-AGA. 4.3 k *CAT8* fragment was amplified from the YS58 genome. The linearized plasmid and *CAT8* fragment were introduced into YS58-Δcat8 strain and homologous recombination resulted in the YCpA-CAT8-EGFP plasmid. *ADH1* promoter and terminator were inserted into the multi-clone sites of YEp352 to obtain YEpA. The YEpA plasmid was linearized, PCR amplified 2.8, 2.2, and 1.5 k *ARO80*, *GLN3*, and *GAT1* fragments from the YS58 genome, and also amplified Myc tag from the commercial plasmid. The linearized plasmid, Myc tag, and gene fragment were mixed and cloned with ClonExpress Ultra One Step Cloning Kit (Vazyme Biotech Co., Ltd., China) to obtain YEpA-ARO80-Myc, YEpA-GLN3-Myc, and YEpA-GAT1-Myc plasmids.

### 2-Phenylethanol Detection

Cell density was tested at *OD*_600_. 2-PE was quantified by HPLC (Agilent 1260 HPLC, United States) equipped with a diode array detector (DAD) using a 250 mm C-18 column (guige; Agilent Technologies, United States). The mobile phase was 50% methanol, the column temperature was 30°C, the flow rate was 1.0 mL/min, the detection wavelength was 206 nm, and the peak time of 2-PE was 7.3 min.

### Microscopic Analysis of Protein Localization

Yeast cells were cultured in YPD until the early log phase (0.8–1 OD), then transferred to 250 ml induction medium containing 2% Glc or Gly for 6 h. In all subsequent experiments, unless specific, the strain culture method was the same as that mentioned here. Fluorescence signals were detected by confocal microscope (Leica SP8), and localization of nuclei was detected by staining DNA with 2.5 μg/ml of 4, 6 diamidino-2-phenylindol (DAPI).

### Western Blotting Analysis

Cells were washed 3 times with cold PBS (pH 7.4) [137 mM NaCl, 2.7 mM KCl, 8 mM Na_2_HPO_4_, and 1.8 mM KH_2_PO_4_], collected by rapid centrifugation at 4°C, and immediately frozen in liquid nitrogen. The cell pellets were ground with liquid nitrogen and added 150 μl PBS [Before using, add 1% PMSF (Thermo Fisher Scientific Inc., United States) and 1% PIC (Beijing Solarbio Science & Technology Co., Ltd., China)] for ice bathing for about 10 min, and then centrifuged at 10,000 rpm for 10 min. The appropriate amount of supernatant was mixed with 5 × SDS loading buffer, boiled for 10 min, and centrifuged at 10,000 rpm for 1 min, supernatants were stored at −80°C. Western blot was performed on 6 or 8% polyacrylamide gels as previously described (Laemmli, 1970; Towbin et al., 1979). Cat8 was detected by incubation with monoclonal anti-HA tag antibody (dilution of 1:5,000; Abcam, United States) and peroxidase-conjugated goat anti-rabbit antibody (dilution of 1:4,000; EASYBIO, China). Aro80, Gln3, and Gat1 were detected by incubation with monoclonal anti-Myc tag antibody (dilution of 1:4,000; Mei5 Biotechnology Co., Ltd., China) and peroxidase-conjugated goat anti-mouse antibody (dilution of 1:3,000; EASYBIO, China). β-Actin was detected by incubation with a β-Actin rabbit polyclonal antibody (dilution of 1:4,000; Mei5 Biotechnology Co., Ltd., China). Bound antibody was detected by using the Pierce ECL Western Blotting Substrate (Thermo Fisher Scientific Inc., United States) as instructed by the manufacturer.

### RNA Preparation and Quantitative RT-PCR (qRT-PCR)

The yeast cells were collected by centrifuging at 4°C and washed with pre-cooled PBS and then crushed by liquid nitrogen grinding. The total RNA was obtained by using the GeneJET RNA Purification Kit (Thermo Fisher Scientific Inc., United States) and the relative amount of specific mRNA was determined by qRT-PCR. Briefly, 2 μg of total RNA was subjected to reverse transcription in a 20 μl 1 × FastKing-RT SuperMix reaction mixture (TIANGEN Biotech Co., Ltd., Beijing, China), and then reacted in 1 × SuperReal PreMix Plus (SYBR Green) reaction mixture (TIANGEN Biotech Co., Ltd., Beijing, China) using Roche LightCycler 96 real-time PCR system (Roche Diagnostics, Germany). Expression data were processed by the second-derivative maximum method of LightCycler 96 software SW1.1. The reference gene was *ACT1* and fold changes were calculated using the 2^–ΔΔCT^ method (Livak and Schmittgen, 2001).

### RNA-Seq, Gene Expression Profiling, and Gene Ontology (GO) Enrichment Analysis

RNA samples were analyzed by RNA-seq (Novogene Co., Ltd., China) on an Illumina NovaSeq 6000 platform (Illumina Inc., United States), and at least 2 Gb clean reads were obtained for each sample. The detailed data was deposited in National Microbiology Data Center (NMDC) with accession numbers NMDC10018022. The FASTX toolkit^1^ was used to evaluate the raw data and discard low-quality reads. The clean reads were aligned to the reference genome of strain S288C using HISAT2 (Pertea et al., 2016), and the numbers of reads mapped to each gene were counted by HTSeq (Anders et al., 2015). The Q30 was > 94% of the reads were mapped to the reference genome. The differentially expressed genes were screened and annotated with DESeq2 (Anders and Huber, 2010; Anders et al., 2015). A corrected *p* ≤ 0.05 and a log2 fold change ≤ −1 or ≥ 1 were set as the threshold for significantly differential expression. GO functional annotation and the KEGG pathway enrichment analysis were performed by the Database for Annotation, Visualization and Integrated Discovery (DAVID) v6.8 (Huang et al., 2009). To understand the relationships between rich clusters, we use Metascape to build the network of selected enriched terms (Zhou et al., 2019).

### ChIP-Seq and Bioinformatic Analysis

Chromatin Immunoprecipitation (ChIP) experiment was conducted as previously described with some modifications (Cam and Whitehall, 2016). The YS58-CAT8-HA strain was cultured in YPD until the early log phase (0.8–1 OD), then transferred to a Phe induction medium containing 2% Glc or Gly for 6 h. Cells were cross-linked with 1% formaldehyde for 20 min and quenched with glycine (final concentration 125 mM) for 15 min at 30°C. Cells were washed 3 times with cold PBS (pH 7.4), quickly frozen in liquid nitrogen, and stored at −80°C for later use. The cell pellet was ground with liquid nitrogen, and 0.6 g of powder was added to 12 ml of pre-chilled ChIP Buffer I [50 mM HEPES-KOH (pH 7.5), 140 mM NaCl, 1% Triton X-100, 1 mM EDTA, and 0.1% Sodium Deoxycholate] [Before using, add 1% PMSF (Thermo Fisher Scientific Inc., United States) and 1% PIC (Beijing Solarbio Science & Technology Co., Ltd., China)] and mixed, and the lysate was divided into six copies into 15 ml of ultrasound tubes, 2 ml each. The chromatin was interrupted by ultrasound with Bioruptor Plus (Diagenode, Belgium), high-power ultrasound for 30 s, and 30 s pause for 1 time, a total of 30 times. The best DNA size after the ultrasound was 250–500 bp. About 20 μl Dynabeads^®^ Protein A (Thermo Fisher Scientific Inc., United States) was washed with ChIP Buffer I, added to the chromatin solution prepared above, and incubated at 4°C for 2–3 h for pre-washing, separated and discarded the magnetic beads. About 500 μl of supernatant was transferred to a new centrifuge tube labeled “input” as the input group and stored at 4°C. 6 μg of monoclonal anti-HA (Abcam, United States) was added to the pre-washed chromatin and incubated overnight at 4°C with rotation, then centrifuged at 12,000 rpm at 4°C for 10 min, and the supernatant was transferred to a new centrifuge tube. The washed magnetic beads (40 μl) were added to the supernatant and incubated at 4°C with rotation for 2–3 h, then the magnetic beads were separated and the supernatant was discarded. Pre-chilled 1 ml ChIP Buffer I, ChIP Buffer II [50 mM HEPES-KOH (pH 7.5), 500 mM NaCl, 1% Triton X-100, 1 mM EDTA, and 0.1% Sodium Deoxycholate], and ChIP Buffer III [10 mM Tris–HCl (pH 8.0), 0.25 M Lithium chloride monohydrate, 1% NP-40, 1 mM EDTA, and 0.1% Sodium Deoxycholate] were used to wash the magnetic beads and each buffer was used to wash the beads at 4°C for 15, 5, and 5 min, respectively. The magnetic beads were washed with 1 ml of TE buffer [100 mM Tris–HCl (pH 8.0) and 10 mM EDTA (pH 8.0)] and the supernatant was discarded. The beads were centrifuged at 3,000 rpm for 1 min at room temperature to remove residual TE buffer. The magnetic beads were incubated with 250 μl Elution buffer [50 mM Tris–HCl (pH 8.0), 10 mM EDTA (pH 8.0), and 1% SDS] at 65°C for 10 min to elute the protein complexes, eluted twice and transferred the supernatant to a new centrifuge tube, labeled “ChIP.” About 20 μl of 5 M NaCl (final concentration 0.2 M) was added to “ChIP” and “input” tubes and incubated overnight at 65°C to decrosslink. High-quality DNA was purified and concentrated by ChIP DNA Clean & Concentrator (Zymo Research Corporation, United States).

The samples were sequenced using the Illumina NovaSeq 6000 platform (Illumina Inc., United States) for high-throughput sequencing (Annoroad Gene Technology Beijing Co., Ltd., China), and the sequencing read length was PE150. A total of 30.8 G data volume was obtained for sequencing, with an average Q30 of 93.25%. The detailed data was deposited in National Microbiology Data Center (NMDC) with accession number: NMDC10018022. The raw data was quality controlled by FastQC, and then Bowtie2 was used for sequence alignment (Langmead and Salzberg, 2012). ChIP peaks were visualized with Integrative Genomics Viewer (IGV) version 2.7.2, a genomic data set viewer for visualization of genomic features (Robinson et al., 2011). We used peak-calling algorithm MACS (Model-based Analysis for ChIP-seq) version 2.2.7.1 (Zhang et al., 2008; Park, 2009), a commonly used peak-caller with more accurate results than competing peak-callers (Thomas et al., 2017). ChIP peaks were annotated by ChIPseeker (Yu et al., 2015), which integrates ChIP annotation, comparison, and visualization and serves as a toolbox for the analysis of ChIP-seq data.

### Co-immunoprecipitation Assay

Co-Immunoprecipitation (Co-IP) was performed as previously described (Björck and Kronvall, 1984; Harlow and Lane, 1988; Wikström et al., 1995), YS58-CAT8-HA Aro80-Myc strain, YS58-CAT8-HA Gln3-Myc strain, and YS58-CAT8-HA Gat1-Myc strain were cultured in YPD until early log phase (0.8–1 OD), then transferred to Phe induction medium containing 2% Gly for 6 h. A total of 50 mg cells were collected, ground with liquid nitrogen and added IP Lysis/Wash Buffer (pH7.4) [25 mM Tris, 150 mM NaCl, 1 mM EDTA, 1% NP-40, and 5% glycerol] 1.5 ml [Before use, add 1% PMSF (Thermo Fisher Scientific Inc., United States) and 1% PIC (Beijing Solarbio Science & Technology Co., Ltd., China)]. After centrifugation at 10,000 rpm for 10 min, lysate supernatant containing 1–2 mg total protein was incubated with 20 μl magnetic beads (washed twice with IP Lysis/Wash Buffer) for pre-cleaning at 4°C for 2 h. The magnetic beads were separated and some of the supernatants were preserved as Input at −20°C. The remaining supernatants were added with an appropriate amount of antibodies and rotated at 4°C for overnight incubation. About 20 μl magnetic beads were added to the supernatant containing antigen-antibody complex and incubated at 4°C for 1 h. Separate the magnetic beads from the supernatant (the supernatant can be preserved until the IP experiment is successful), then wash the magnetic beads with 1 ml IP Lysis/Wash Buffer three times rotating for 10 min at 4°C each time. The magnetic beads were transferred to a new centrifugal tube and 500 μl Saline Solution [150 mM NaCl] was added to clean the magnetic beads for 10 min at 4°C. The separated magnetic beads were mixed with 50 μl 2 × SDS PAGE Sample Buffer [100 mM Tris (pH 6.8), 40 mM DTT, 2% SDS, 20% glycerol, and 0.2% bromophenol blue] and boiled for 5–8 min at 100°C. The supernatant was taken and the isolated binding protein was analyzed by Western blot.

### Mass Spectrometry and Post-translational Modification Analysis

The proteins were digested with trypsin and subjected to an EASY-nLC 1000 interfaced via a Nanospray Flex ion source to an Orbitrap Fusion Tribrid mass spectrometer (Thermo Fisher Scientific, United States) (nano-LC-MS/MS) analysis at the Technological Platform of Mass Spectrum Centre of Institute of Microbiology, Chinese Academy of Sciences. The peptides were loaded onto a trap column (C18, 3 μm particles, 100 μm ID, 3 cm length, Dr. Maisch GmbH) and separated using an analytical column (C18, 1.9 μm particles, 150 μm ID, 15 cm length, Dr. Maisch GmbH) at a flow rate of 500 nl/min with a 60 min LC gradient composed of Solvent A (0.1% formic acid) and Solvent B (acetonitrile, 0.1% formic acid). The gradient was 3–8% B for 5 min, 8–22% B for 40 min, 22–35% B for 10 min, 35–90% B for 3 min, and finally 90% B for 2 min. The mass spectrometer was operated in a data-dependent acquisition mode, in which the precursor MS1 scan (m/z 350–1,550) was acquired in the Orbitrap at a resolution setting of 120,000, followed by Orbitrap HCD-MS/MS and OTHCD-MS/MS of the 20 most abundant multiply charged precursors in the MS1 spectrum. MS2 spectra were acquired at a resolution of 30,000.

MS/MS data was processed using Mascot search engine (v.2.8.0, 2021^2^; Matrix Science Ltd., London, United Kingdom). Tandem mass spectra were searched against protein sequence, and Trypsin/P was specified as a cleavage enzyme allowing up to two missing cleavages. For precursor ions, the mass error was set to 10 ppm, and for fragment ions, the mass error is set to 0.02 Da. Carbamidomethylation on Cys was specified as a fixed modification and oxidation on Met and Phosphorylation on Ser and Thr were specified as variable modifications. False discovery rate thresholds for protein, peptide, and modification sites were specified at 1%. All the other parameters in Mascot were set to default values.

## Results

### Overexpressed Cat8 Enhances 2-PE Production by Improving Cell Growth in the Presence of Glucose

**FIGURE 1:**
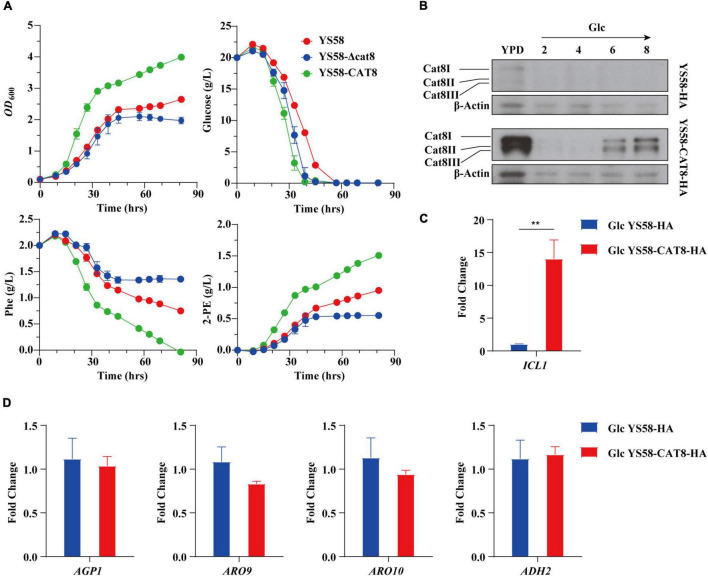
Effect of Cat8 on 2-PE biosynthesis and the transcriptional activity of Cat8 in the presence of glucose. **(A)** Cell growth, residual sugar, Phe, and 2-PE contents. Yeast strains were cultured in fermentation medium at 30°C and 200 rpm. **(B)** Cat8 level assay. Cells were cultured in YPD overnight and transferred to fermentation medium with glucose as the only carbon sources for 6 h at 30°C and 200 rpm. Samples were taken at indicated times. Cell free extracts (2 μg protein) were subjected to immunoblot analysis using polyclonal anti-HA and β-Actin antibodies. **(C)** Transcriptional levels of Cat8 target gene *ICL1*. **(D)** Transcriptional levels of genes involved in Ehrlich pathway. For **(C,D)**, yeast cells were cultured in fermentation medium with glucose as the only carbon source at 30°C and 200 rpm for 6 h. Data shown are mean values ± SDs of triplicates. Statistical difference was determined by two-tailed Student’s *t*-test, **p < 0.01.

We characterized the related fermentation phenotypes of the wild-type strain YS58, *CAT8* knock-out strain (YS58-Δcat8), and *CAT8* overexpression strain (YS58-CAT8) in the fermentation medium (Figure 1A). In the overexpression strain, strong constitutive promoter pTEF2 was used in order to obtain the stable and high-level transcription of *CAT8*. The cell growth, 2-PE yield, and glucose and Phe consumption of YS58-CAT8 were higher than those of YS58 and YS58-Δcat8 during the whole growth process. To fully understand the role of Cat8 in the biosynthesis of 2-PE, the protein levels of Cat8 and its transcriptional activation activity were determined. A significant increase in Cat8 levels was observed in strain YS58-CAT8-HA compared to the control strain YS58-HA, confirming the enhanced expression of Cat8 (Figure 1B). The transcriptional levels of *ICL1*, a key target gene of Cat8 in the glyoxylic acid pathway (Rahner et al., 1999) in strain YS58-CAT8-HA were 13-fold higher than that in strain YS58-HA (Figure 1C), suggesting that constitutively overexpressed Cat8 possess transcriptional activation activity under glucose condition. mRNA levels of genes such as amino acid permease gene *AGP1*, transaminase gene *ARO9*, decarboxylase gene *ARO10*, and reductase gene *ADH2* had no significant differences between strain YS58-CAT8-HA and YS58-HA at the early stage of exponential growth under glucose condition (Figure 1D), indicating that enhanced 2-PE in glucose medium might result mainly from increased cell growth.

### Snf1 Is Essential for Cat8 Nucleic Localization and Enhances the Regulatory Activity of Cat8

**FIGURE 2:**
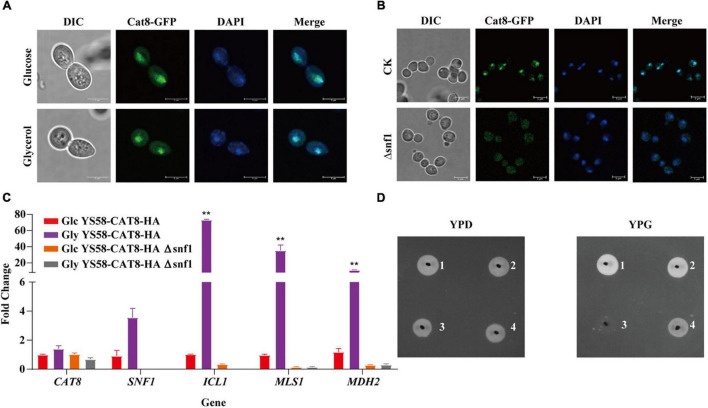
Cat8 intracellular localization and transcriptional regulatory activity in response to different carbon sources. **(A)** Cat8 intracellular localization under glucose or glycerol condition. **(B)** Effect of Snf1 on Cat8 subcellular localization under glycerol condition. For **(A,B)**, the white bar represents 5 μm. **(C)** Transcriptional levels of genes *CAT8*, *SNF1*, *ICL1*, *MLS1*, and *MDH2* normalized to *ACT1*. **(D)** Growth assay to indicate the effect of Snf1 deletion and Cat8 overexpression, YS58-HA (1), YS58-CAT8-HA (2), YS58-HA Δ snf1 (3), and YS58-CAT8-HA Δ snf1 (4). Data shown are mean values ± SDs of triplicates. Statistical difference was determined by two-tailed Student’s *t*-test, **p < 0.01.

To explore the role of Cat8 in the regulation of 2-PE synthesis, different carbon sources were further adopted, and the subcellular localization of Cat8 was detected. GFP-tagged Cat8 was mainly concentrated in the nucleus whether fermented carbon source glucose or non-fermented carbon source glycerol was the sole carbon source (Figure 2A). However, the deletion of protein kinase Snf1 caused the accumulation of Cat8 in the cytoplasm even under glycerol conditions (Figure 2B). In our constitutively overexpressed Cat8 strain, Snf1 might be active but with weak activity under glucose conditions, and Cat8 was phosphorylated to a low degree. The transcriptional levels of Cat8 target genes including *ICL1*, *MLS1*, and *MDH2* in glycerol conditions were 5, 36, and 10 times higher than those in glucose conditions, respectively, while their mRNA levels decreased significantly in *SNF1* knock out strain (Figure 2C), indicating the partial activity of Cat8 under glucose condition and the significance of Snf1 for the Cat8 activity. Strain YS58-HA Δsnf1 could not use glycerol to grow, while strain YS58-CAT8-HA Δsnf1 showed a weak phenotype of glycerol utilization (Figure 2D), suggesting that when Cat8 overexpressing in Snf1 deletion strain, other kinases might participate in the phosphorylation of Cat8, which were weak and not the central kinases for Cat8 compared to Snf1.

### Cat8 Plays an Essential Role in Response to External Nutritional Changes

**FIGURE 3:**
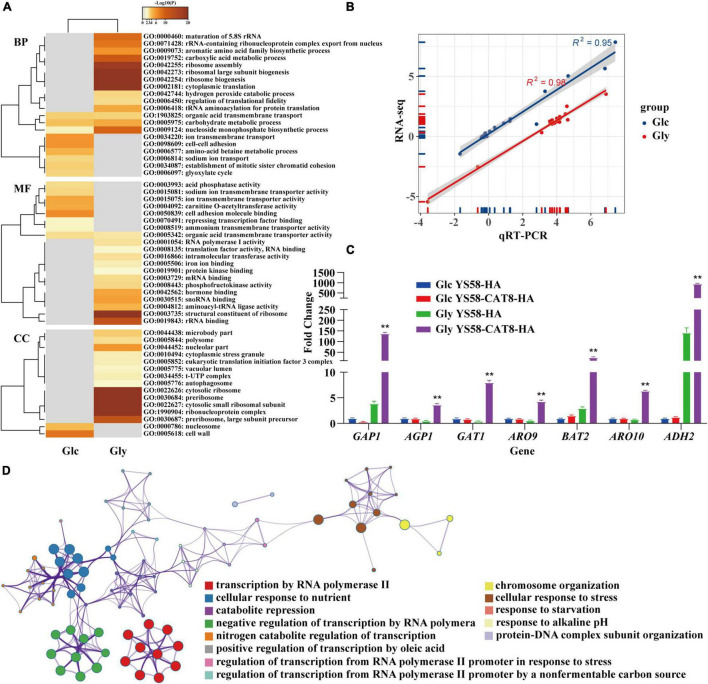
RNA-seq analysis of yeast strain in response to external nutritional changes. **(A)** The list of differential genes in glucose (Glc) and glycerol (Gly) after overexpression of Cat8 was enriched by GO differential heat map of biological process (BP), molecular function (MF), and cellular component (CC). Heatmap showing the top enrichment clusters, one row per cluster, using a discrete color scale to represent statistical significance. Gray color indicates a lack of significance. **(B)** The correlation between RNA-seq and qRT-PCR results. **(C)** The transcriptional levels of genes related to fusel alcohols synthesis via Ehrlich pathway in RNA-seq data. **(D)** Metascape visualization of the interactome network formed by all 78 gene candidates from differential expression of transcription factors after Cat8 overexpression under Gly condition, where each node represented an enriched term and was colored by its cluster-ID. The strains used were YS58-HA and YS58-CAT8-HA. Data shown are mean values ± SDs of triplicates. Statistical difference was determined by two-tailed Student’s *t*-test, **p < 0.01.

RNA-seq was performed to explore the regulatory network of Cat8 in response to Phe induction under glucose or glycerol condition. Gene expression heat map showed that the two carbon sources greatly influenced the transcript spectrums of yeast cells after Cat8 overexpression. The transcription levels of 2.3% of genes on a genome-wide scale changed more than two times between the wild-type strain and *CAT8* overexpressing strain under glucose conditions, while the differential genes accounted for 28.8% of the total genes under glycerol conditions (Supplementary Figure 2), which was consistent with the changes of more than 1,700 genes during the diauxic shift of *S. cerevisiae* reported by DeRisi et al. (1997). Gene Ontology (GO) analysis showed that Cat8 overexpression enhanced the TCA cycle, respiratory metabolism, and glyoxylic acid cycle rather than metabolic pathways related to 2-PE synthesis under glucose conditions. In the TCA cycle, the transcription of isocitrate lyase gene *ICL1*, cytoplasmic malate dehydrogenase gene *MDH2*, isocitrate dehydrogenase gene *IDP2*, and malate synthase gene *MLS1* were up-regulated 32.8-fold, 2.7-fold, 2.4-fold, and 2.1-fold, respectively, (Figure 3A and Supplementary Dataset 1). Under glycerol conditions, activities of various metabolic pathways, such as glycerol metabolism, respiratory metabolism, fatty acid metabolism in the peroxisome, amino acids (arginine, proline, and phenylalanine) catabolism, were enhanced by overexpressing *CAT8*, while glycolysis, gluconeogenesis, biosynthesis of amino acids (lysine, phenylalanine, tyrosine, and tryptophan) were inhibited (Figure 3A). Moreover, a variety of transmembrane transport pathways including amino acid transport, ammonium transport, ion transport, and drug transport were activated in *CAT8* overexpressing strain (Figure 3A and Supplementary Dataset 2), which is partly related to substrate uptake and efflux of by-products in the process of 2-PE synthesis.

About 17 actively expressed genes under glucose or glycerol condition were randomly selected and determined their transcription levels by qRT-PCR. A strong correlation between RNA-seq and qRT-PCR for the transcription levels of 17 genes was observed (Figure 3B and Supplementary Table 3), indicating the reliability of RNA-seq results. Among the 17 genes, the transcription of amino acid permease genes *GAP1* and *AGP1*, transcriptional activator gene *GAT1*, Ehrlich pathway genes *ARO9*, *BAT2*, *ARO10*, and *ADH2* in *CAT8* overexpressing strain were up-regulated 36-fold, 7-fold, 18-fold, 7-fold, 9-fold, 8-fold, and 7-fold, respectively, under glycerol condition compared to that of the wild-type strain (Figure 3C and Supplementary Table 3). The transcription levels of *PDR12* and *ESBP6* for fusel acids export increased by 2.3-fold and 3.4-fold after Cat8 overexpression (Supplementary Table 3) in glycerol conditions. These results suggested that highly active Cat8 in *S. cerevisiae* not only activated carbon metabolism-related pathways but also enhanced the transcriptional levels of key enzymes in the Ehrlich pathway, upstream amino acid transporters, downstream metabolites fusel acid transporters, and transcriptional activator Gat1, showing that Cat8 regulation on 2-PE synthesis is comprehensive and extensive.

The up-regulated genes in Cat8 overexpressing strain under glycerol conditions were enriched in many terms related to “sequence-specific DNA binding,” “transcriptional activator activity,” and “sequence-specific DNA binding of RNA polymerase II regulatory region” in the molecular function analysis of GO. Based on the quantitative measurement of functional similarity of terms (Huang et al., 2009) and using Metascape website to analyze the pathways and processes of the genes in the above terms, 13 significantly enriched (*p* < 0.01) GO terms were obtained (Figure 3D and Supplementary Dataset 3) forming different enrichment networks. The results showed that highly active Cat8 was involved in regulating cell response to carbon and nitrogen sources under glycerol conditions. In terms of carbon sources, Cat8 mainly participated in regulating non-fermenting carbon source utilization and fatty acid metabolism; in terms of nitrogen sources, Cat8 was involved in derepressing nitrogen catabolite repression (NCR).

### Cat8 Regulates the Transcription of Genes Related to Carbon and Nitrogen Metabolism by Direct Binding With Promoters

**FIGURE 4:**
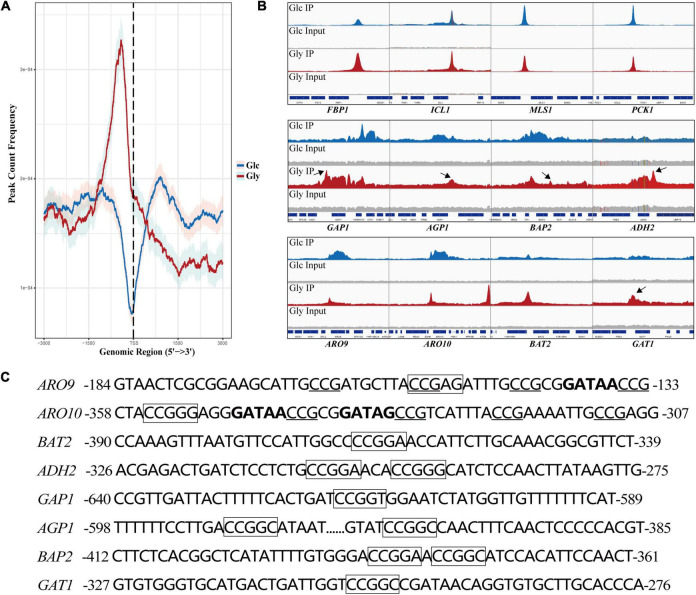
Analysis of binding peaks of transcription factor Cat8 under two carbon sources by ChIP-seq. **(A)** The distribution of reads in the peak region (3,000 bp upstream and downstream of TSS) on all transcripts under the glucose (Glc) or glycerol (Gly) condition was calculated to reflect the binding intensity of Cat8 to DNA at 0.95 confidence interval. **(B)** IGV examined the peaks enriched by ChIP-seq. The blue and red peaks represented the binding peaks of Cat8 under the conditions of Glc and Gly, respectively, and the gray was input. **(C)** Promoter sequences of Cat8 target genes include *ARO9*, *ARO10*, *BAT2*, *ADH2*, *GAP1*, *AGP1*, *BAP2*, and *GAT1*. Cat8 binding sites (CCGGN) were marked with a box. CCG triplets, the binding site of Aro80, were underlined, and GATA factor binding sites (GATAA/G) were indicated in bold.

To explore the interaction of Cat8 and its target genes, chromatin immunoprecipitation sequencing (ChIP-seq) was carried out for strain YS58-CAT8-HA under glucose or glycerol conditions with Phe induction for 6 h. Cat8 was mainly bound to the upstream 1,000 bp region from the transcription start site (TSS) of target genes, and the binding ability was significantly increased under glycerol conditions compared to glucose conditions (Figure 4A). Using Integrative Genomics Viewer (IGV) visualization tool, the binding peaks of Cat8 and ChIP-seq sequencing readings in the gene promoter region were analyzed. The results showed that the binding peak map of Cat8 with *ARO9* and *ARO10* related to nitrogen metabolism was similar to that with *FBP1*, *ICL1*, *MLS1*, and *PCK1* involved in carbon metabolism (Figure 4B). The motif of Cat8 target genes for non-fermentative carbon source utilization was CCGGN (Badis et al., 2008). We combined the central sequence of binding peaks displayed by IGV to determine the binding elements of Cat8. As a result, the transcriptional binding sites of Cat8 on the key enzyme genes *ARO9*, *BAT2*, *ARO10*, and *ADH2* in the Ehrlich pathway, amino acid transporter genes *GAP1*, *AGP1*, and *BAP2*, and transcriptional activator *GAT1* were identified, whose sequences and respective positions were fairly coincident with the above-reported motif (Figure 4C).

Analysis of Cat8 interacting genes showed that there were 380 genes under glucose conditions with 209 unique binding genes, while there were 458 genes directly interacting with Cat8 under glycerol conditions with 287 unique binding genes (Supplementary Figure 3). Interestingly, we noticed that a large number of Cat8 binding peaks could be gotten under glucose conditions, which might be due to the low level of phosphorylation of Cat8 by weak Snf1 activity or by other kinases in Cat8 overexpression strain. In the presence of glycerol, Cat8 activity was enhanced by the phosphorylation catalyzed by completely active Snf1 to make Cat8 bind with much more genes, resulting in sharper and higher binding peaks than that under glucose conditions (Figure 4B). In addition, we noticed that there were prominent Cat8 binding peaks in the promoter region of some genes. However, the transcriptional levels of these genes were down-regulated under glucose and glycerol conditions (Supplementary Dataset 4), which might be due to the competition between Cat8 and other transcription activators for the same *cis*-regulatory element. Most of the previous studies explored Cat8 regulation on carbon metabolism under glucose limitation. However, according to our transcriptome data and ChIP-seq data, Cat8 had transcriptional activation function when Cat8 was constitutively strongly expressed under glucose conditions, inferring that the Snf1 signal pathway might be partially activated; it was also speculated that other kinases in yeast cells might phosphorylate Cat8.

Furthermore, Cat8 binding genes under two conditions were enriched by GO (BP\CC\MF) and KEGG (Supplementary Dataset 5), and the enrichment network was also analyzed (Supplementary Figure 4). The result is consistent with that in our transcriptome data analysis, which confirmed again that transcriptional activator Cat8 could not only regulate carbon metabolism but also play an important role in responding to nitrogen starvation and regulating cellular nitrogen metabolism. Biosynthesis regulation was dominant under glucose conditions, while catabolism regulation was prominent under glycerol conditions.

### Cat8 Interacts Directly With Transcription Factor Aro80 and GATA Activating Factor Gat1

**FIGURE 5:**
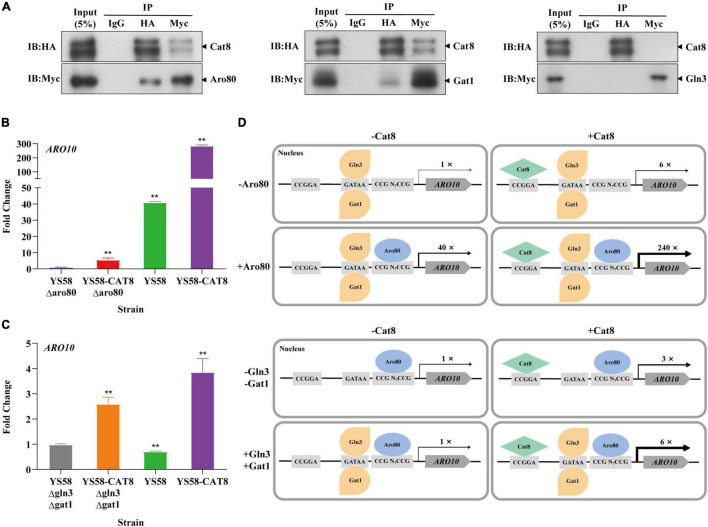
The interaction of Cat8 with Aro80 and GATA activating factors. **(A)** The Co-immunoprecipitation of total protein extracted from strains YS58-CAT8-HA Aro80-Myc, YS58-CAT8-HA Gat1-Myc, and YS58-CAT8-HA Gln3-Myc from left to right. Input (5%) was used as a positive control and IgG as a negative control. Immunoprecipitated proteins (IPs) were analyzed by immunoblotting and probing with HA and Myc antibodies. **(B)** Effect of Aro80 on the *ARO10* transcription level of wild-type strain and *CAT8* overexpression strain. **(C)** Effect of GATA factors on the *ARO10* transcription level of wild-type strain and *CAT8* overexpression strain. **(D)** The interaction pattern of Cat8 with Aro80 and GATA activator factors. Yeast cells were cultivated in fermentation medium with glycerol as the only carbon source for 6 h. *ACT1* was used as the reference gene, and wild-type strains with *ARO80* or *GLN3*/*GAT1* deletion were used as controls. Data shown are mean values ± SDs of triplicates. Statistical difference between strains was determined by two-tailed Student’s *t*-test, **p < 0.01.

Based on the analysis of ChIP-seq, it was found that the binding sites of Cat8 on the key enzyme genes *ARO9*/*ARO10* of the Ehrlich pathway were close to those of transcriptional activator Aro80 and GATA activators Gln3 and Gat1 (Figure 4C). We speculated that Cat8 might interact with them and coordinately regulate gene transcription. To detect the intracellular interaction of Cat8 with Aro80, Gln3, and Gat1, the above three proteins with Myc tags were overexpressed in the HA tagged Cat8 strain (YS58-CAT8-HA), then Co-Immunoprecipitation (Co-IP) assay for the protein complexes with anti-HA, anti-Myc, and IgG antibodies, respectively, were carried out. The results showed protein-level interaction between Cat8 and Aro80 and Gat1, while there was no such interaction between Cat8 and Gln3 (Figure 5A).

The transcription level of *ARO10* was analyzed by qRT-PCR after deleting Aro80 in the wild-type strain and Cat8 overexpression strain. The results showed that the *ARO10* expression levels in yeast cells with Aro80 deletion decreased to 2.5% of that without Aro80 deletion, indicating that Aro80 was the main transcription factor for activating *ARO10*, which was consistent with the previous report on Aro80 (Lee and Hahn, 2013). However, in Cat8 overexpression strain, the transcriptional level of *ARO10* increased six-fold regardless of whether Aro80 deletion or not (Figure 5B). This indicated that Cat8 itself could activate transcription of *ARO10* in the absence of Aro80. When Aro80 exists, the interaction between Cat8 and Aro80 may make them bind to the promoter region of *ARO10* more tightly and bring a significant increase in the transcription of *ARO10* (Figure 5D).

There is no significant difference in *ARO10* level between the wild-type strain and the strain with GATA activator Gln3 and Gat1 deletion (Figure 5C). It had been reported that when yeast cells grew in YPD medium to the early logarithmic stage and were treated with rapamycin, the *ARO10* transcription level in GATA activator deletion strains decreased by 50% (Lee and Hahn, 2013). The inconsistency between this study and the literature may be caused by the different cultural conditions or strain differences. When Cat8 was overexpressed in Gln3 and Gat1 deletion strain, the *ARO10* level increased by three times. When Gln3 and Gat1 existed, Cat8 overexpression enhanced *ARO10* transcription by six times. The difference between the above two group strains was two times. Considering the Co-IP results, it was suggested that the interaction and coordination between Cat8 and Gat1 are also involved in the transcriptional regulation of *ARO10*.

## Discussion

Cat8 is a crucial factor for non-fermentable carbon source utilization. During the analysis of growth and 2-PE synthesis, we found that diauxic growth disappeared in YS58-Δcat8, in the meantime, the 2-PE titer in this strain did not change either at the later stage, hinting that the synthesis of 2-PE is coupled with cell growth. Combined with the transcriptome data, we speculated that overexpression of Cat8 increases the metabolic activity of the cellular TCA cycle and the glyoxylate cycle under glucose conditions, this enabled continuous active production of α-ketoglutarate (the direct precursor to glutamate), and glutamate is the end product of the biosynthetic nitrogen source (Butow and Avadhani, 2004); furthermore, α-ketoglutarate is an intermediate receptor for the α-amino generated by transamination in the Ehrlich pathway (Etschmann et al., 2002). This allows cells to obtain higher biomass and 2-PE yield.

Cat8 relies on phosphorylation to play its function, and Snf1 is the main kinase of Cat8. It was reported that Snf1 phosphorylating Cat8 was inhibited in the presence of glucose and could not activate Cat8 in the wild-type yeast (Busti et al., 2010; Turcotte et al., 2010). However, overexpressed Cat8 in our study played the role in the whole process of yeast cell growth, and the results of RNA-seq and ChIP-seq also showed that overexpressed Cat8 could bind to the target gene and activate the related pathway under the glucose condition. Recently, some researchers found that an unusual ratio of nutrients could trigger some signal pathways in yeast resulting in unexpected activation of Snf1 under high reducing sugars conditions (Vallejo et al., 2020). Hence, we surmised that when Cat8 was constitutively overexpressed, the Snf1 signal pathway might be triggered and Snf1 was partially activated to phosphorylate Cat8 at a low level. The constitutive overexpression of *CAT8* also can restore the phenotype of the snf1-deletion strain that originally cannot use non-fermented carbon sources. This implies that there may be other kinases involved in the phosphorylation of Cat8 in yeast, which might be weaker and not the main kinases for Cat8 compared with Snf1.

The residue site Ser562 was reported to be the key phosphorylation site of Cat8 directly acted by Snf1 (Charbon et al., 2004), while the SGD database contains 13 of the Cat8 phosphorylation sites reported in the previous literature (Albuquerque et al., 2008; Holt et al., 2009; Swaney et al., 2013; Lanz et al., 2021) but does not contain Ser562, suggesting that differences of phosphorylation sites in Cat8 might exist in different strains. In this study, three bands of Cat8I∼III were presented after Western Blot (Supplementary Figure 5A). Mass spectrometry detection showed the differences in post-translational modification (PTM) such as phosphorylation, methylation, and acetylation among these three bands. For example, phosphorylation modification at Thr53 or Ser55, methylation at Arg84, and acetylation at Lys507 and Lys936 were different (Supplementary Table 4). The strain in our research did not show Ser562 phosphorylation either, further reflecting different key phosphorylation sites in Cat8 in different yeast strains. During Western Blot analysis, we also found the differences in Cat8 bands pattern (Supplementary Figures 5B,C) compared to that in the previous report (Randez-Gil et al., 1997), for example, the Cat8 band pattern did not change after the Lambda-phosphatase treatment. Amino acid sequence alignment showed quite a few differences in the sequences between our strain and the previously reported strain, including multiple amino acid site mutations and a fragment deletion/insertion (InDel) (Supplementary Figure 6A). Selecting the reported Cat8 protein sequences of *S. cerevisiae* in various habitats for phylogenetic analyses, it was found that the strains used in the previous literature (CEN.PK2-1C) and in this study (YS58) were located in the wild/transitional group and liquid-state fermentation group (LSF), respectively (Supplementary Figure 6B), which further confirmed the above differences.

The domesticated lineages of yeast *S. cerevisiae* documented worldwide so far belong to two major monophyletic groups associated with solid-and liquid-state fermentation (SSF and LSF), respectively (Duan et al., 2018). Interestingly, we note that the molecular phylogenetic tree constructed from Cat8 protein sequences clearly distinguishes between the LSF and the SSF in the domestication lineages. Previous studies on Cat8 mainly focused on the regulation of carbon source metabolism (Turcotte et al., 2010), and this study proposed that Cat8 also plays a regulatory role in nitrogen source metabolism such as amino acids catabolism. Perhaps the obvious difference in the Cat8 protein sequence among different strains is due to adaptive evolution. These results imply that Cat8 may play an essential role in *S. cerevisiae*’s adaptation to different fermentation types as a global transcriptional regulator, and may further help the yeast better adapt to different nutritional environments during the evolutionary process through phosphorylation, acetylation, methylation, and other modifications.

The combined analysis of RNA-seq and ChIP-seq of overexpression Cat8 in fermentative and non-fermentative carbon sources conditions provided large-scale information on cell reprogramming responses to nutritional changes. Cat8 regulates carbon metabolism, such as activating the glyoxalic acid cycle and gluconeogenesis pathway and simultaneously participates in responding to nitrogen hunger and regulates nitrogen metabolism. Overexpression of Cat8 activated multiple transcription factors, most of which participated in the cellular response to nutrients. More Cat8 binding peaks were pulled under glycerol conditions, and the peak type was sharper and taller than that under glucose conditions. Combined with the results of Figure 4A, we speculated that the difference in binding peaks might be related to the phosphorylation of Cat8 on the one hand; On the other hand, it may be related to the level of cell autophosphorylation. When cells are exposed to non-fermentative carbon sources, the Snf1 phosphokinase complex is activated completely, and transcriptional activation is promoted through the activation of transcription factors and chromatin structure reconstruction of the target gene promoter region (Agricola et al., 2004; Tachibana et al., 2005; Biddick et al., 2008; Broach, 2012). Therefore, Cat8 has a strong ability to bind DNA under glycerol conditions. These results suggest that Cat8 has a more comprehensive regulation in nutritional response, and plays a similar role as a global regulator of energy homeostasis or an inhibitor of the anabolic process. Cat8 can regulate the transcription of target genes in different metabolic pathways through sensing carbon source signals to control the direction of cell metabolism.

In this study, it was demonstrated that Cat8 directly binds and regulates key enzyme genes of the Ehrlich pathway (*ARO9*, *BAT2*, *ARO10*, and *ADH2*), amino acid transporter gene (*GAP1* and *AGP1*), and transcriptional activator (*GAT1*). At the same time, the results proved that Cat8 interacts with GATA activator Gat1 and Ehrlich pathway-specific transcriptional activator Aro80 at the protein level, and the level of transcription activation of target genes between them can be superimposed on each other. The nature of transcription factors’ protein-protein interactions and their affinity for DNA binding motifs define gene target selectivity and chromatin binding dynamics (Francois et al., 2020). Therefore, the binding of the transcription factors to the promoter region is more stable, which improves the transcription level of the target gene. This study indicates that Cat8 directly regulates the critical steps of yeast synthesis of 2-PE, which helps to improve the efficiency of 2-PE synthesis. It has been suggested that Gat1 plays a core role in NCR control, and small changes in Gat1 expression have a high impact on the expression of NCR genes (Georis et al., 2009). Cat8 not only regulates the transcription of Gat1 but also interacts with Gat1. Therefore, Cat8 overexpression may participate in the regulation of nitrogen metabolism by regulating the GATA factor and making cells respond to starvation. In addition, we found that overexpression of Cat8 resulted in obvious cell flocculation (results not shown) and enrichment in the pathways related to flocculation and cell adhesion. Flocculation was related to stress resistance (Soares, 2011), which is also a coping method for yeast to adapt to the changes in different environments.

In summary, Cat8 participates in both non-fermentative carbon and nitrogen utilization. In the regulation of aromatic alcohol synthesis, Cat8 can promote Ehrlich pathway flux by directly binding and activating the related genes, it also can interact with other transcription factors such as Aro80 and Gat1 to coordinate enhance the Ehrlich pathway. As a global transcription factor, Cat8 plays a significant role in response to nutritional changes and regulates both carbon and nitrogen metabolism.

## Data Availability Statement

The data presented in the study are deposited in the National Microbiology Data Center (NMDC) repository, accession number NMDC10018022, available at the link: https://nmdc.cn/resource/genomics/sra?keyword=NMDC10018022.

## Author Contributions

ZD: designing experiments, implementation, data curation, and writing – original draft, review and editing HD: experiments implementation and data curation. YC, ZZ, and XG: methodology, data analysis, and experimental assistance. ZW: conceptualization, supervision, funding acquisition, and writing – original draft, review and editing. XH: conceptualization, supervision, and writing – review and editing All authors agreed to be accountable for the content of the work.

## Conflict of Interest

The authors declare that the research was conducted in the absence of any commercial or financial relationships that could be construed as a potential conflict of interest.

## Publisher’s Note

All claims expressed in this article are solely those of the authors and do not necessarily represent those of their affiliated organizations, or those of the publisher, the editors and the reviewers. Any product that may be evaluated in this article, or claim that may be made by its manufacturer, is not guaranteed or endorsed by the publisher.

## Footnotes

1
https://github.com/agordon/fastx_toolkit/

2
http://www.matrixscience.com

## Supplementary Material

Supplementary Dataset 1 Results of differential gene analysis under Glc and Gly conditions (transcriptome data).

Supplementary Dataset 2 Results of GO enrichment analysis under Glc and Gly conditions (transcriptome data).

Supplementary Dataset 3 Information and GO enrichment results of 78 selected transcription factors.

Supplementary Dataset 4 Enrichment rate and fold change of Cat8 binding genes under Glc and Gly conditions.

Supplementary Dataset 5 Enrichment analysis results of GO and KEGG pathways of Cat8 binding genes under Glc and Gly conditions (ChIP-seq data).
