# Supplementary material for: Cat8 Response to Nutritional Changes and Interaction With Ehrlich Pathway Related Factors
Source: Front Microbiol. 2022 Jun 15;13:898938. doi: 10.3389/fmicb.2022.898938 (PMC9245043; doi:10.3389/fmicb.2022.898938)
Supplement: Supplementary file 1 [file Data_Sheet_1.docx]

# **
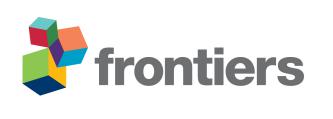
1 Supplementary Tables**

**Table S1. List of strains used in this study**

| **Strains** | **Complete genotype** | **References or sources** |
| --- | --- | --- |
| *S. cerevisiae* YS58 | MATα flo1 ura3-52 leu2-3 112 his4-519 trp1-789 | (Teunissen et al., 1993) |
| YS58-HA | MATα flo1 ura3-52 leu2-3 112 his4-519 trp1-789 CAT8:3HA:*Sh ble* | This study |
| YS58-CAT8-HA | MATα flo1 ura3-52 leu2-3 112 his4-519 trp1-789 *NEO*:Ptef2:CAT8:3HA:*Sh ble* | This study |
| YS58-HA ∆snf1 | MATα flo1 ura3-52 leu2-3 112 his4-519 trp1-789 CAT8:3HA:*Sh ble* snf1Δ | This study |
| YS58-CAT8-HA ∆snf1 | MATα flo1 ura3-52 leu2-3 112 his4-519 trp1-789 *NEO*:Ptef2:CAT8:3HA:*Sh ble* snf1Δ | This study |
| YS58-CAT8 | MATα flo1 ura3-52 leu2-3 112 his4-519 trp1-789 Ptef2:CAT8 | This study |
| YS58-∆cat8 | MATα flo1 ura3-52 leu2-3 112 his4-519 trp1-789 cat8∆ | This study |
| YS58-∆aro80 | MATα flo1 ura3-52 leu2-3 112 his4-519 trp1-789 aro80∆ | This study |
| YS58-∆gln3gat1 | MATα flo1 ura3-52 leu2-3 112 his4-519 trp1-789 gln3∆ gat1∆ | This study |
| YS58-CAT8 ∆aro80 | MATα flo1 ura3-52 leu2-3 112 his4-519 trp1-789 aro80∆ Ptef2:CAT8 | This study |
| YS58-CAT8 ∆gln3∆gat1 | MATα flo1 ura3-52 leu2-3 112 his4-519 trp1-789 gln3∆ gat1∆ Ptef2:CAT8 | This study |
| CAT8-EGFP | MATα flo1 ura3-52 leu2-3 112 his4-519 trp1-789 cat8∆/YCpA-CAT8-EGFP | This study |
| CAT8-EGFP Δsnf1 | MATα flo1 ura3-52 leu2-3 112 his4-519 trp1-789 cat8∆/YCpA-CAT8-EGFP snf1Δ | This study |
| YS58-CAT8-HA Aro80-Myc | MATα flo1 ura3-52 leu2-3 112 his4-519 trp1-789 *NEO*:Ptef2:CAT8:3HA:*Sh ble* /YEpA-ARO80-Myc | This study |
| YS58-CAT8-HA Gln3-Myc | MATα flo1 ura3-52 leu2-3 112 his4-519 trp1-789 *NEO*:Ptef2:CAT8:3HA:*Sh ble* /YEpA-GLN3-Myc | This study |
| YS58-CAT8-HA Gat1-Myc | MATα flo1 ura3-52 leu2-3 112 his4-519 trp1-789 *NEO*:Ptef2:CAT8:3HA:*Sh ble* /YEpA-GAT1-Myc | This study |

**Table S2 List of plasmids used in the study**

| **Plasmids Name** | **Genotype** | **References or sources** |
| --- | --- | --- |
| pGMZC | YEp352 with P*_GAL1_*-mazF-T*_AOX1_* and P*_TEF1_*-zeoR-T*_CYC1_* | (Zhou et al., 2021) |
| pYC-AGA | *ADH1* promoter and terminator and GFP cloned into YCp50 (HindIII/EcoRI) | (Zhou et al., 2021) |
| pFA6a-kanMX4 | E. coli-Yeast shuttle plasmid (Amp^r^ and G418^r^) | (Wach, 1996) |
| pAG32 | E. coli plasmid (Amp^r^ and Hyg^r^) | (Goldstein and McCusker, 1999) |
| YCpA-*CAT8*-EGFP | The plasmid containing ADH1p-*CAT8*-EGFP-ADH1t | This study |
| YEp352 | E. coli-Yeast shuttle plasmid (Amp^r^ and *URA3*) | (Hill et al., 1986) |
| YEpA | *ADH1* promoter and terminator cloned into YEp352 (HindIII/EcoRI) | (Chen et al., 2017) |
| pUC57-Myc | E. coli plasmid (Amp^r^ and Myc tag) | This study |

**Table S3.** **Lists of genes used in quantitative real-time PCR analysis and quality of the data obtained**

| **Gene** | **Glucose** | | **Glycerol** | |
| --- | --- | --- | --- | --- |
|  | **qRT-PCR(log2 FC mean)** | **transcriptome(log2 FC mean)** | **qRT-PCR(log2 FC mean)** | **transcriptome(log2 FC mean)** |
| AGP1 | -0.107430768 | 0.018271585 | 4.010249442 | 1.236193422 |
| ARO2 | -0.193425968 | 0.277890104 | -0.641065768 | -2.520046034 |
| ARO4 | -0.424134432 | -0.118304662 | -3.575264975 | -5.434315896 |
| ADH2 | 0.061336475 | 0.429685837 | 3.612982007 | 1.117853297 |
| ARO9 | -0.38260026 | -0.019868754 | 3.597973257 | 1.271443126 |
| ARO10 | -0.265733991 | 0.107935371 | 3.732757205 | 1.364109235 |
| BAT2 | 0.354763982 | 0.74692635 | 4.196272688 | 1.516724701 |
| CAT8 | 7.437494144 | 7.853083577 | 4.17571545 | 1.647284396 |
| GAT1 | -0.276679773 | -0.061441933 | 4.594325536 | 2.517300228 |
| GAP1 | -1.674303275 | -1.446610561 | 6.890452426 | 3.529664579 |
| ICL1 | 4.671501342 | 5.036756916 | 3.858438928 | 1.108578428 |
| IDP2 | 1.239646289 | 1.283732729 | 3.113689071 | 0.33090516 |
| MLS1 | 0.890271812 | 1.102888437 | 4.163513891 | 1.209287739 |
| MDH2 | 1.274174963 | 1.451193444 | 3.719995368 | 1.041495103 |
| SFC1 | 6.82889926 | 5.661030139 | 4.644721866 | 1.39216382 |
| SIP4 | 2.812768901 | 1.030139167 | 4.001174355 | 1.510472497 |
| YAT1 | 3.31163534 | 3.764091123 | 4.511886362 | 1.875239295 |
| PDR12 | NA | 0.140610179 | NA | 1.179213671 |
| ESBP6 | NA | 0.352091259 | NA | 1.760627369 |

**Table S4. The Phosphorylation, Methylation, and Acetylation modification sites in Cat8I and Cat8II, III**

| Protein bands | Modified type | Sequences of peptides | Modified site |
| --- | --- | --- | --- |
| Cat8I | Phosphorylation modification | TASPTPLSTPIYR | Thr53 or Ser55 |
|  |  | DAPLNLSSTNIYLLNQTVNK | Ser173 or Ser174 or Thr175 |
|  |  | NNLSFNNKSNYSLTK | Ser1167 and Ser1170 |
|  | Methylation modification | RPQCSQCAAVGFECR | Arg84 |
|  |  | LLALCDIK | Lys137 |
|  | Acetylation modification | LMGLPPATTTSLKPLFGSQSK | Lys936 |
| Cat8 II、III | Phosphorylation modification | DAPLNLSSTNIYLLNQTVNK | Ser173 or Ser174 or Thr175 |
|  |  | NNLSFNNKSNYSLTK | Ser1167 and Ser1170 |
|  | Methylation modification | LLALCDIK | Lys137 |
|  | Acetylation modification | LMAYYHQLSLIIPK | Lys507 |

The red font represents modification sites.

# **2 Supplementary Figures**


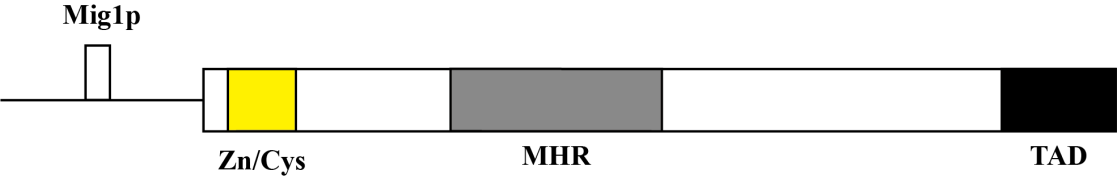


**Supplementary Figure 1** Schematic diagram of *CAT8* functional domain. Zn(2)-Cys(6) binuclear cluster domain (Zn/Cys, yellow); the middle homology region (MHR, grey); the C-terminus required for carbon source-dependent transcriptional activation (TAD, black); the Mig1p binding site (box).


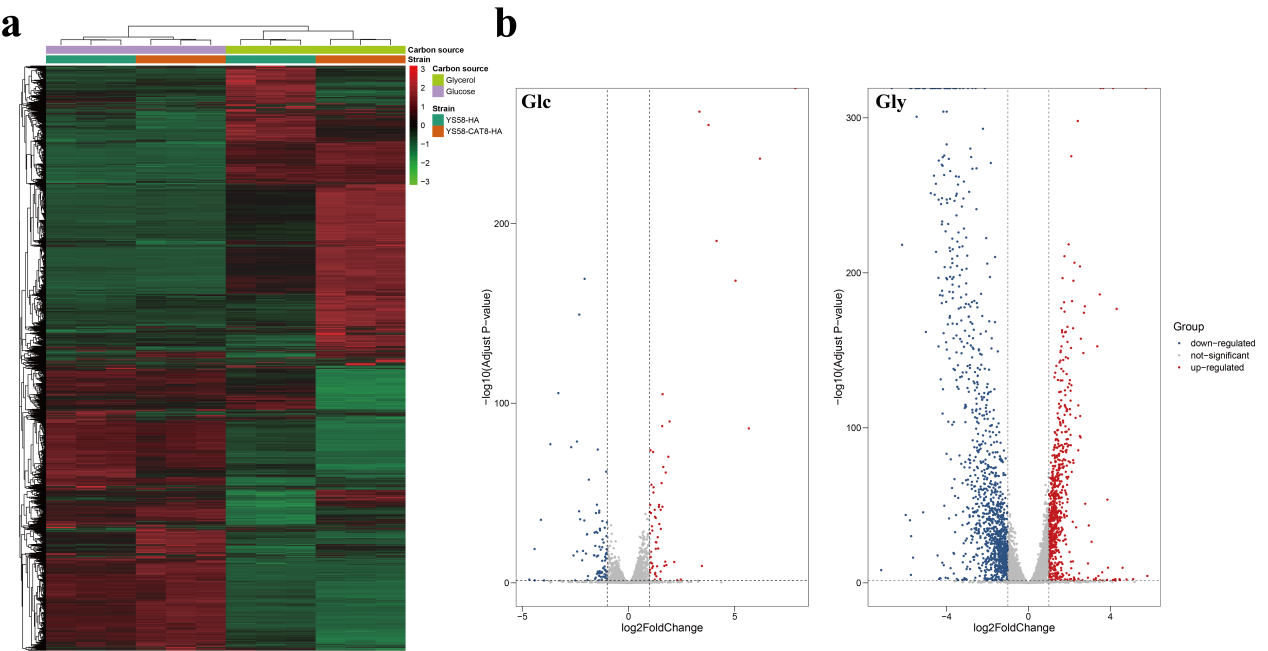


**Supplementary Figure 2** Changes of global gene expression level after Cat8 overexpression in *Saccharomyces cerevisiae* under two carbon sources.

1. Hierarchical clustering and heat map of gene expression level of YS58-HA and YS58-CAT8-HA strains under two kinds of carbon sources. The rows represent genes and the columns represent samples. The colors of the annotations distinguish between carbon sources and strains. Red indicate high expression, green low, black intermediate.
2. Differential gene volcano plots of Cat8 overexpression under two carbon sources (fold change >2 and *P*<0.05). The vertical lines correspond to 2.0-fold up and down (log2 ratio), and the horizontal line represents a *P*-value of 0.05. So the red or blue dots in the plot represent the up-regulated or down-regulated differential genes with statistical significance. Under Glc condition, 62 genes were up-regulated and 90 genes were down-regulated. Under Gly condition, 763 genes were up-regulated and 1149 genes were down-regulated.


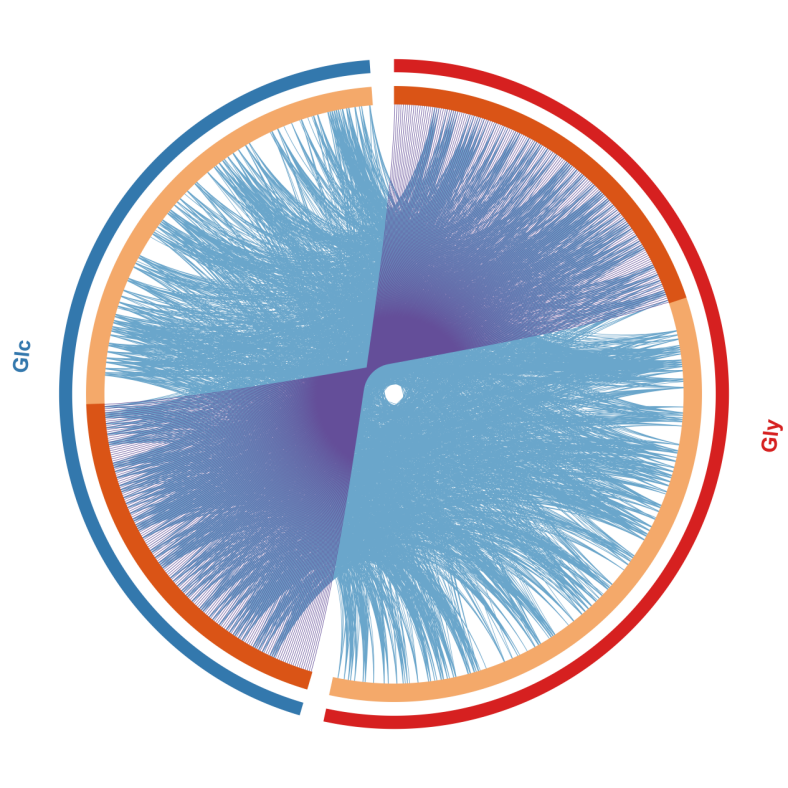


**Supplementary Figure 3** The overlap of Cat8 binding genes based on gene function or shared pathway under the condition of Glc and Gly.

Blue and red arcs were used to represent Cat8 binding genes under Glc and Gly conditions, respectively. The genes bound by Cat8 under both carbon sources were expressed in dark orange, while the unique binding genes were expressed in light orange.


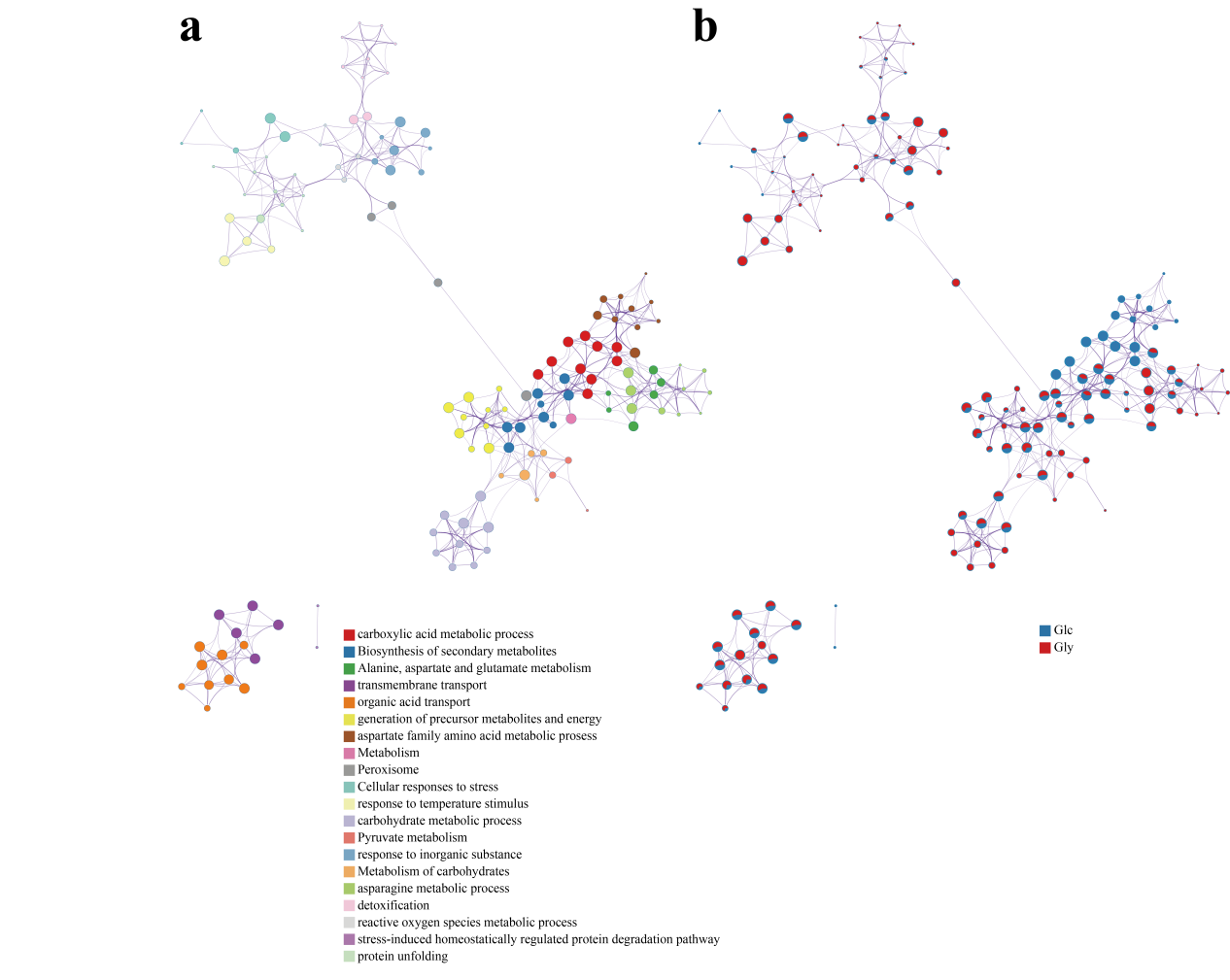


**Supplementary Figure 4** Functional enrichment network of Cat8 binding genes under Glc and Gly conditions.

1. Metascape website was used for analysis, the network is visualized using Cytoscape7, terms enriched by Cat8 binding genes under Glc and Gly conditions were taken as nodes in the network. The functionally similar terms was grouped into a cluster and distinguished by color, where terms with a similarity > 0.3 were connected by edges, and presented as a network graph. Here we shown 20 clusters with the best p value (*P*<0.01).
2. The network structure was consistent with figure a, and the pie chart showed the proportion of Cat8 binding genes under two carbon sources in each term.


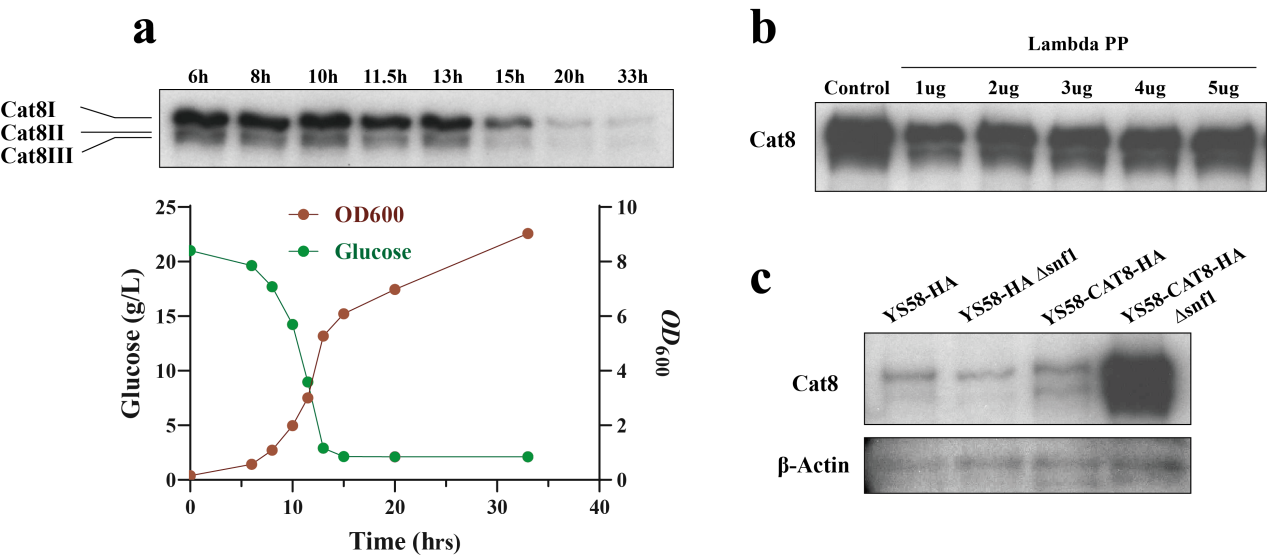


**Supplementary Figure 5** Electrophoresis migration analysis of Cat8 protein.

1. YS58-CAT8-HA strain was inoculated in YPD medium to an OD_600_ of 0.01 and the contents of residual sugar in supernatant and the *OD*_600_ were monitored for 34 h, samples were taken at the indicated times. Proteins (2mg per lane) were separated by SDS-PAGE in 8% polyacrylamide and subjected to immunoblot analysis using polyclonal anti-HA antibody.
2. Protein extracts from YS58-CAT8-HA strain were treated with Lambba-phosphatase. SDS-PAGE and immunoblot analysis were as indicated for Figure S5a. Cells were grown under YPD medium with 2% glucose to the mid-log phase. Phosphatase reactions were done on 10mg of total protein at 37℃ for 1 h. The amount of Lambda-phosphatase added is indicated above each lane, and nothing was added to the control.
3. Cells were grown under YPD medium with 2% glucose to 24 h. Proteins (2mg per lane) were separated by SDS-PAGE and analyzed by immunoblotting as indicated for Figure 1B.


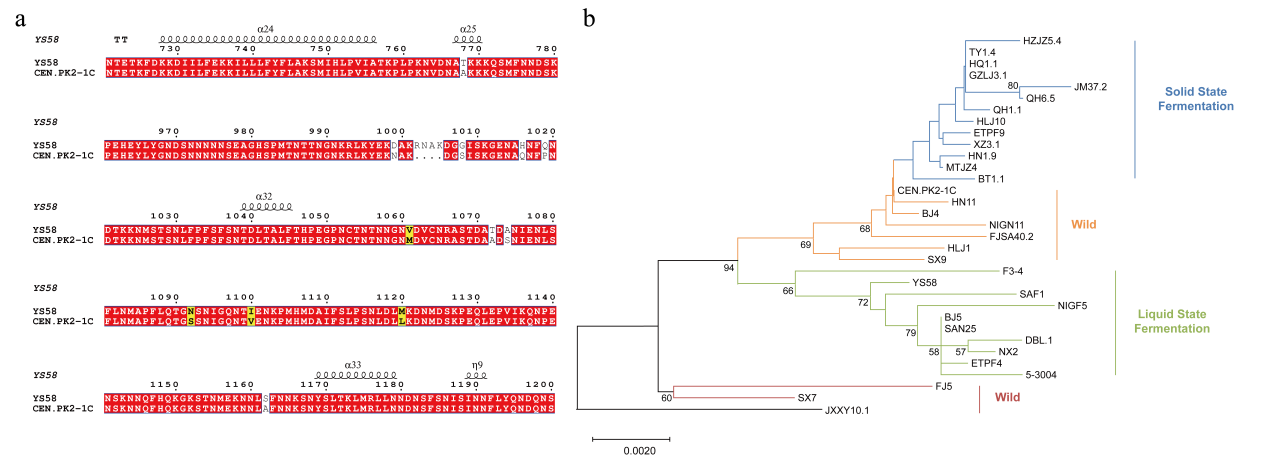


**Supplementary Figure 6** **Molecular analysis of the Cat8 protein sequence and phylogenetic tree of Cat8 proteins in *S. cerevisiae* under different habitats**

1. Numbers indicated the position of the amino acid in each line of the proteins within the corresponding full-length protein sequence. Protein sequence alignment was carried out using the method as in the literature (Robert and Gouet, 2014). ENDscript displays full-length protein sequence alignment of Cat8. The red background indicates the consensus site, and the yellow and white background indicates the difference site.
2. Phylogenetic tree of Cat8 proteins in *S. cerevisiae* under different habitats. The phylogenetic tree was constructed using the neighbor-joining method by the MEGAX software. The reliability of the trees was tested using a bootstrapping method with 1000 replicates. Numbers indicated bootstrap values for 1000 replicates. Blue and green indicate that the strains were isolated from solid and liquid fermentation environments, respectively, while yellow and red indicate that the strains were isolated from the wild.

# **3 Supplementary Reference**

Chen, X., Wang, Z., Guo, X., Liu, S., and He, X. (2017). Regulation of general amino acid permeases Gap1p, GATA transcription factors Gln3p and Gat1p on 2-phenylethanol biosynthesis via Ehrlich pathway. J Biotechnol. 242, 83-91. doi:10.1016/j.jbiotec.2016.11.028

Goldstein, A. L., and McCusker, J. H. (1999). Three new dominant drug resistance cassettes for gene disruption in *Saccharomyces cerevisiae*. Yeast. 15(14), 1541-1553. doi:10.1002/(SICI)1097-0061(199910)15:14<1541::AID-YEA476>3.0.CO;2-K

Hill, J. E., Myers, A. M., Koerner, T. J., and Tzagoloff, A. (1986). Yeast/*E. coli* shuttle vectors with multiple unique restriction sites. Yeast. 2(3), 163-167. doi:10.1002/yea.320020304

Robert, X., and Gouet, P. (2014). Deciphering key features in protein structures with the new ENDscript server. Nucleic Acids Res. 42, W320-W324.

Teunissen, A. W., van den Berg, J. A., and Steensma, H. Y. (1993). Physical localization of the flocculation gene *FLO1* on chromosome I of Saccharomyces cerevisiae. Yeast. 9(1), 1-10. doi:10.1002/yea.320090102

Wach, A. (1996). PCR-synthesis of marker cassettes with long flanking homology regions for gene disruptions in *S. cerevisiae*. Yeast. 12(3), 259-265. doi:10.1002/(SICI)1097-0061(19960315)12:3%3C259::AID-YEA901%3E3.0.CO;2-C

Zhou, C., Li, M., Lu, S., Cheng, Y., Guo, X., He, X., et al. (2021). Engineering of cis-element in *Saccharomyces cerevisiae* for efficient accumulation of value-added compound squalene via downregulation of the downstream metabolic flux. J Agric Food Chem. 69(42), 12474-12484. doi:10.1021/acs.jafc.1c04978
